# Identification and Characterization of Genomic Predictors of Sarcopenia and Sarcopenic Obesity Using UK Biobank Data

**DOI:** 10.3390/nu15030758

**Published:** 2023-02-02

**Authors:** Ekaterina A. Semenova, Erinija Pranckevičienė, Elvira A. Bondareva, Leysan J. Gabdrakhmanova, Ildus I. Ahmetov

**Affiliations:** 1 Department of Molecular Biology and Genetics, Federal Research and Clinical Center of Physical-Chemical Medicine of Federal Medical Biological Agency, 119435 Moscow, Russia; 2 Research Institute of Physical Culture and Sport, Volga Region State University of Physical Culture, Sport and Tourism, 420138 Kazan, Russia; 3 Department of Human and Medical Genetics, Biomedical Science Institute, Faculty of Medicine, Vilnius University, LT-01513 Vilnius, Lithuania; 4 Department of Systems Analysis, Faculty of Informatics, Vytautas Magnus University, LT-44404 Kaunas, Lithuania; 5 Laboratory of Genetics of Aging and Longevity, Kazan State Medical University, 420012 Kazan, Russia; 6 Department of Physical Education, Plekhanov Russian University of Economics, 115093 Moscow, Russia; 7 Research Institute for Sport and Exercise Sciences, Liverpool John Moores University, Liverpool L3 5AF, UK

**Keywords:** sarcopenia, sarcopenic obesity, genetics, DNA, nutrition, physical activity, testosterone, height, fat-free mass, weakness

## Abstract

The substantial decline in skeletal muscle mass, strength, and gait speed is a sign of severe sarcopenia, which may partly depend on genetic risk factors. So far, hundreds of genome-wide significant single nucleotide polymorphisms (SNPs) associated with handgrip strength, lean mass and walking pace have been identified in the UK Biobank cohort; however, their pleiotropic effects on all three phenotypes have not been investigated. By combining summary statistics of genome-wide association studies (GWAS) of handgrip strength, lean mass and walking pace, we have identified 78 independent SNPs (from 73 loci) associated with all three traits with consistent effect directions. Of the 78 SNPs, 55 polymorphisms were also associated with body fat percentage and 25 polymorphisms with type 2 diabetes (T2D), indicating that sarcopenia, obesity and T2D share many common risk alleles. Follow-up bioinformatic analysis revealed that sarcopenia risk alleles were associated with tiredness, falls in the last year, neuroticism, alcohol intake frequency, smoking, time spent watching television, higher salt, white bread, and processed meat intake; whereas protective alleles were positively associated with bone mineral density, serum testosterone, IGF1, and 25-hydroxyvitamin D levels, height, intelligence, cognitive performance, educational attainment, income, physical activity, ground coffee drinking and healthier diet (muesli, cereal, wholemeal or wholegrain bread, potassium, magnesium, cheese, oily fish, protein, water, fruit, and vegetable intake). Furthermore, the literature data suggest that single-bout resistance exercise may induce significant changes in the expression of 26 of the 73 implicated genes in m. vastus lateralis, which may partly explain beneficial effects of strength training in the prevention and treatment of sarcopenia. In conclusion, we have identified and characterized 78 SNPs associated with sarcopenia and 55 SNPs with sarcopenic obesity in European-ancestry individuals from the UK Biobank.

## 1. Introduction

Sarcopenia is an age-associated condition characterized by the loss of skeletal muscle strength and muscle mass, and in severe cases, followed by reduced physical performance (e.g., slower gait speed) [1]. In most cases (53–84%), sarcopenia co-exists with obesity [2,3]. Older adults are usually identified as having sarcopenic obesity if low muscle mass and strength, as well as increased adiposity are present [4,5,6]. Furthermore, individuals with sarcopenic obesity can be stratified into stage I (absence of clinical complications) or stage II (presence of clinical complications) [7].

The prevalence of sarcopenia and sarcopenic obesity depends on the diagnostic criteria used to describe these conditions, with the range from 10–27% for sarcopenia [6], and from 10–23% for sarcopenic obesity [8]. Both sarcopenia and sarcopenic obesity are related to negative health-related outcomes, such as increased risk of falls, disability, frailty, osteoporosis, type 2 diabetes (T2D), metabolic syndrome, poor glycaemic profiles (i.e., hyperglycaemia, high HbA1c, insulin resistance, etc.) cardiovascular diseases, dyslipidaemia, poor neurocognitive functioning and quality of life, decreased health span and mortality [9,10,11,12,13,14]. Importantly, while declines in lean mass could contribute to further gains in fat mass, high fat mass may also lead to accelerated loss of lean mass [10].

There is a wide range of individual variability in skeletal muscle quantity and quality even under the same biological (age, sex) and environmental (level and type of physical activity, macro and micronutrient intake, etc.) conditions. This variability partly depends on genetic factors, with high heritability for muscle strength (49–56%) [15] and fat-free mass (45–76%) [16]. So far, hundreds of genome-wide significant (*p* < 5 × 10^−8^) single nucleotide polymorphisms (SNPs) associated with sarcopenia-related traits, such as handgrip strength (170 SNPs) [17,18,19], appendicular lean mass (1059 SNPs) [20] and walking pace (70 SNPs) [21] have been individually identified in the UK Biobank cohort. However, their pleiotropic effects on all three traits have not been investigated on a systemic level.

Since muscle strength has been shown to be positively correlated with muscle mass and walking speed [22,23], we hypothesized that these phenotypes might be positively associated at a genetic level as well. One might also suggest that alleles associated with all three sarcopenia-related traits (i.e., low muscle strength, low lean mass and slow walking pace) can be considered as the most robust predictors of sarcopenia. On the other hand, risk alleles for both sarcopenia and increased adiposity can be considered as genomic predictors of sarcopenic obesity.

The aims of the present study were threefold: (1) to identify SNPs with pleiotropic effects on handgrip strength, appendicular lean mass, usual walking pace and fat percentage using summary statistics from the UK Biobank cohort; (2) to identify the potential mechanism of action of each SNP on sarcopenia-related traits by searching for intermediate phenotypes and using mice knockout models; and (3) to investigate the effect of resistance exercise on the expression of sarcopenia-related genes using bioinformatic tools.

## 2. Materials and Methods

### 2.1. UK Biobank Study

The UK Biobank is an open-access large prospective study with phenotypic and genotypic data from more than 500,000 participants (>90% of participants are of white ethnicity) with an age range for inclusion of 40–69 years when recruited in 2006–2010 [24]. UK Biobank has approval from the North West Multi-centre Research Ethics Committee (MREC) as a Research Tissue Bank (RTB) approval (reference 11/NW/0382). Full written informed consent was obtained from all participants prior to the study.

### 2.2. Identification of Genomic Predictors of Sarcopenia and Sarcopenic Obesity Using UK Biobank Data

In the first stage, we used publicly available summary statistics from five published genome-wide association studies (GWASes) on handgrip strength [17,18,19], appendicular lean mass [20] and walking speed [21], making an initial list of 1299 genome-wide significant (*p* < 5 × 10^−8^) SNPs (Table 1). To identify correspondences between phenotypes (for example, to find if a specific SNP associated with handgrip strength is also associated with appendicular lean mass and walking pace), we used publicly available summary statistics from GWASes on appendicular lean mass [20], appendicular lean mass in older adults [25], handgrip strength (left) [26], handgrip strength (right) [26], handgrip strength in older adults (weakness) [19] and usual walking pace [26] with less stringent *p* value threshold (*p* < 0.005) (Table 1). SNPs associated with all three phenotypes (i.e., with appendicular lean mass, handgrip strength and walking pace) with consistent effect directions were considered as potential genomic predictors of sarcopenia.

In the second stage, to test the hypothesis that sarcopenia-related SNPs are also associated with other health-related traits, we used summary statistics from GWASes on body fat percentage [26], type 2 diabetes [26], heel bone mineral density [27], frequency of tiredness [26], self-reported tiredness [26], recent feelings of tiredness or low energy [26], and falls in the last year [26] (Table 1). Risk alleles for both sarcopenia and increased body fat percentage were considered as genomic predictors of sarcopenic obesity. Furthermore, risk alleles for both sarcopenic obesity and type 2 diabetes were considered as genomic predictors of sarcopenic diabesity. We also used other traits (biochemical, anthropometric, physiological, behavioral) to identify shared genetic architecture between sarcopenia-related traits and lifestyle exposures (Table 1).

### 2.3. Analysis of Sarcopenia-Related Polygenic Profiles in European Populations

Raw genetic data of sarcopenia-related SNPs of 503 anonymized individuals of European origin from the 1000 Genomes project (Phase 3) [35] were used to calculate a genetic sum score of risk alleles for each individual. This cohort was composed of five subgroups (British from England and Scotland (*n* = 91), Finnish in Finland (*n* = 99), Toscani in Italia (*n* = 107), Iberian populations in Spain (*n* = 107), Utah residents (CEPH) with Northern and Western European ancestry (*n* = 99). Unweighted polygenic risk scores (coded as 0, 1 and 2 for homozygous non-risk genotype, heterozygous genotype and homozygous risk genotype, respectively) were developed for the prediction of sarcopenia, sarcopenic obesity and sarcopenic diabesity in European populations. Individuals were evenly divided into 5 groups (20% each) with high (high number of risk alleles), above average, average, below average and low (low number of risk alleles) risks for sarcopenia, sarcopenic obesity and sarcopenic diabesity.

### 2.4. Analysis of Association of Sarcopenia-Related SNPs with Gene Expression

The Genotype-Tissue Expression (GTEx) portal [36] was used to analyze the association between sarcopenia-related SNPs and expression of genes in different tissues with the focus on skeletal muscle tissue and nervous system (*p* < 0.05). The GTEx project is an ongoing effort to build a comprehensive public resource to study tissue-specific gene expression and regulation. Samples were collected from 49 tissue sites across >800 individuals, primarily for molecular assays including whole genome sequencing (WGS), whole exome sequencing (WES), and RNA-Seq [37]. SNPs that were significantly (*p* < 0.05) correlated with expression of genes (levels of mRNAs) were considered as expression quantitative trait loci (eQTLs).

### 2.5. Analysis of Effects of Knockouts of Implicated Genes on Sarcopenia-Related Traits in Mice

Data from the International Mouse Phenotyping Consortium (IMPC) database [38] were used to assess the effects (*p* < 0.05) of genes knockout on lean mass, fat mass and grip strength in mice. The IMPC web portal makes available curated, integrated and analyzed knockout mouse phenotyping data from 9000 mouse lines [39].

### 2.6. Analysis of Effects of Strength Training on the Expression of Sarcopenia-Related Genes

Publicly available human skeletal muscle transcriptome dataset was used to check the significant effect (*p* < 0.05) of a single-bout resistance exercise on the mRNA of the sarcopenia-related genes in m. vastus lateralis of seven young men (age 23.3  ±  0.6 years) at 2.5 h and 5 h timepoints compared to baseline [40].

## 3. Results

### 3.1. Potential Genomic Predictors of Sarcopenia and Sarcopenic Obesity

**Figure 1:**
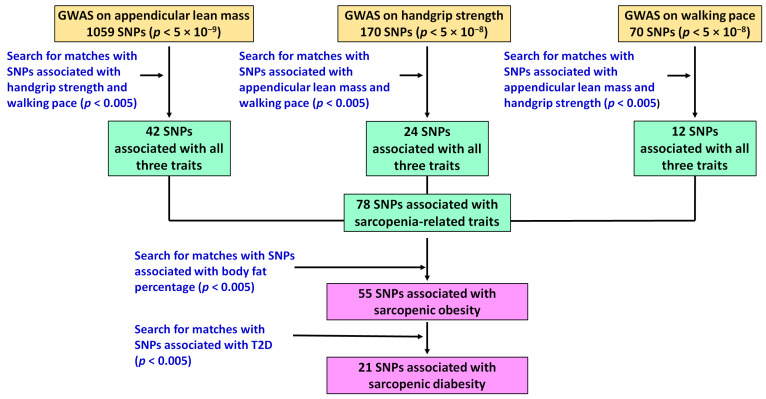
A schematic overview of the study design and the main findings.

A flow diagram displaying the study design and the main findings is shown in Figure 1. First, by combining data from five published GWASes on handgrip strength [17,18,19], appendicular lean mass [20] and walking speed [21], we made a list of 1299 genome-wide significant SNPs. The 78 out of those SNPs (from 73 loci) were independently (i.e., all SNP with linkage disequilibrium (LD) threshold r^2^ > 0.2 were excluded) associated (*p* < 0.005) with all three traits—appendicular lean mass, handgrip strength and walking pace—with consistent effect directions (Table 2). These 78 SNPs can be considered as potential genomic predictors of sarcopenia and can be used as instruments for Mendelian randomization analysis to study and uncover causal relationships between sarcopenia and other traits.

Next, by using summary statistics from GWASs on other health-related traits we found that 55 of the 78 SNPs were associated with body fat percentage with consistent effect directions (i.e., the same allele is a risk variant for both sarcopenia and adiposity) and can be regarded as potential genomic predictors of sarcopenic obesity. Of these 55 SNPs, the 21 SNPs were also associated with the risk of T2D with consistent effect directions (potential genomic predictors of sarcopenic diabesity) (Table 2).

Follow-up bioinformatic analysis revealed that sarcopenia risk alleles were associated with tiredness (16 SNPs), falls in the last year (8 SNPs), neuroticism (12 SNPs), alcohol intake frequency (24 SNPs), smoking (14 SNPs), time spent watching television (22 SNPs), higher salt (8 SNPs), white bread (14 SNPs), and processed meat (6 SNPs) intake. In contrast, the protective alleles were positively associated with bone mineral density (10 SNPs), serum testosterone (23 SNPs), IGF1 (3 SNPs), and 25-hydroxyvitamin D (9 SNPs) levels, height (69 SNPs), intelligence (25 SNPs), cognitive performance (20 SNPs), educational attainment (45 SNPs), income (26 SNPs), physical activity (26 SNPs), ground coffee drinking (12 SNPs) and healthier diet (muesli (17 SNPs), cereal (7 SNPs), wholemeal or wholegrain bread (12 SNPs), potassium (4 SNPs), magnesium (4 SNPs), cheese (18 SNPs), oily fish (11 SNPs), protein (3 SNPs), water (4 SNPs), fruit (16 SNPs), and vegetable (7 SNPs) intake) (Supplementary Table S1).

### 3.2. Polygenic Analysis of Sarcopenia, Sarcopenic Obesity and Sarcopenic Diabesity

A genetic sum score of sarcopenia risk alleles composed of 78 SNPs was calculated for each of 503 individuals of European origin from the 1000 Genomes project. Individuals were then evenly (by ~20%) divided into five groups. Carriers of 58–68 risk alleles had the lowest risk of sarcopenia, whereas carriers of 81–95 risk alleles had the highest risk. The distribution of risk alleles in each subgroup for sarcopenia, sarcopenic obesity and sarcopenic diabesity is shown in Table 3 and may be used to improve prediction of these disease states when incorporated into existing clinical risk tools in individuals of European origin.

### 3.3. Association of Sarcopenia-Related SNPs with Expression of Genes

Of the 78 SNPs, the 58 were identified as eQTL SNPs in the GTEx database that correlated with expression of genes in various tissues including skeletal muscle (32 SNPs), nervous system (13 SNPs), testis (3 SNPs), thyroid (3 SNPs), adipose (1 SNP), left ventricle (3 SNPs), digestive system (2 SNPs), and artery (1 SNP) (Supplementary Table S1).

### 3.4. Effects of Gene Knockouts of Implicated Genes on Sarcopenia-Related Traits in Mice

As mentioned above, the discovered 78 SNPs are located in or near 73 genes. Using IMPC database we assessed the effects of genes knockout on lean mass, fat mass and grip strength in mice. Data for the knockout effects of 35 genes (of the 73 genes) were available in the database, of which 27 were significant (*p* < 0.05). Knockouts in 12 genes (*Adcy3*, *Aoc1*, *Bckdhb*, *Btnl2*, *Cdkal1*, *Cep192*, *Gdf5*, *H1fx*, *Pold3*, *Rbl2*, *Swt1*, *Znf462*) led to the decrease in lean mass and strength (with increase in fat mass), whereas knockouts in 15 genes (*Adpgk*, *Btrc*, *Camkmt*, *Dipk1a*, *E2f3*, *Foxp1*, *Htt*, *Igf2bp3*, *Jmjd1c*, *Lcorl*, *Mllt10*, *Mtch2*, *Ncoa1*, *Piezo1*, *Trib1*) had positive effects on lean mass and strength, but negative on fat mass (Supplementary Table S1).

By comparing human (GWAS, GTEx) and mice genes knockout data, we identified eight genes with the same direction of association. More specifically, while protective alleles in *ADCY3* (rs10203386 T), *BCKDHB* (rs9350850 C), *CEP192* (rs1786263 G), *H1FX* (rs4073154 G), and *POLD3* (rs72977282 T) genes were associated with the increased expression of these genes in human tissues, the knockout of the corresponding genes in mice (*Adcy3*, *Bckdhb*, *Cep192*, *H1fx*, and *Pold3*) led to the decrease in lean mass and strength (with increase in fat mass).

On the other hand, while protective alleles in *BTRC* (rs10883618 A), *LCORL* (rs1472852 C), *MTCH2* (rs11039324 G) genes were associated with a decreased expression of these genes in human tissues, the knockout of the corresponding genes in mice (*Btrc*, *Lcorl*, and *Mtch2*) led to the increase in lean mass and strength (with decrease in fat mass) (Supplementary Table S1).

### 3.5. Effects of Strength Training on the Expression of Sarcopenia-Related Genes

Publicly available human skeletal muscle transcriptome dataset [40] provides evidence on the effects of a single-bout resistance (*n* = 7) exercise on the mRNA expression in 73 implicated genes. Using this data, we found that strength training induces significant changes in the expression of 26 genes (10 upregulated: *ADCY3*, *E2F3*, *JMJD1C*, *JUND*, *MLN*, *MYO1C*, *PIEZO1*, *PPARD*, *SFMBT1*, *ZNF462*; 16 downregulated: *CDKAL1*, *CEP192*, *DLEU1*, *GADD45G*, *GBF1*, *GLCCI1*, *MAML3*, *MMS22L*, *NYAP2*, *SDCCAG8*, *SWT1*, *TRIB1 WWP2*, *XPO4*, *ZBTB38*, *ZNF420*) (Supplementary Table S1), which may partly explain beneficial effects of strength training in the prevention and treatment of sarcopenia.

## 4. Discussion

In this study, we identified and characterized 78 pleiotropic genomic predictors of sarcopenia based on previously discovered genome-wide significant SNPs associated with handgrip strength, appendicular lean mass and walking pace. Of the 78 SNPs, 55 polymorphisms were also associated with body fat percentage and 25 polymorphisms with type 2 diabetes (T2D), indicating that sarcopenia, obesity and T2D share many common risk alleles. It is, therefore, unsurprising that according to the Data from Health and Nutrition Examination Survey (NHANES), 83.6% of women and 79.3% of men (aged 60 and older) with sarcopenia also have obesity (i.e., sarcopenic obesity) [2].

The identified 78 SNPs are located in or near 73 genes that have multiple functions including apoptosis (*GLCCI1*, *JUND*), calcium signaling (*CAMKMT*), carbohydrate metabolism (*ADPGK*, *CDKAL1*, *GIP*, *PRRC2A*), DNA repair (*MMS22L*, *POLD3*), growth and development (*AOC1*, *CEP192*, *DLEU1*, *FHL2*, *FKBPL*, *GADD45G*, *GDF5*, *IGF2BP3*, *IL11*, *JARID2*, *LCORL*, *LIN28A*, *NPPC*, *PIEZO1*, *PITX1*, *PKDCC*, *POU6F2*, *SDCCAG8*, *TRIB1*, *VCAN*), immune surveillance (*BTNL2*, *HLA-DRB1*, *HLA-DRB5*), intracellular transport (*GBF1*, *KIF1B*, *RIN3*, *SLC39A8*, *XPO4*), lipid metabolism (*ADCY3*, *E2F3*, *HMGA2*, *MTCH2*, *NCOA1*, *NMT1*, *PPARD*), myogenesis (*SFMBT1*), neurogenesis (*DIPK1A*, *FOXP1*, *HTT*, *NYAP2*, *ZBTB38*, *ZNF568*), protein metabolism (*BCKDHB*, *BTRC*, *COMMD4*, *SERPINA1*, *WWP2*), regulation of translation (*CELF4*), signal transduction (*SOCS5*), smooth muscle contraction (*MLN*), and transcriptional regulation (*H1FX*, *HABP4*, *JMJD1C*, *MAML3*, *MLLT10*, *MYO1C*, *NCL*, *PML*, *RBL2*, *SOX5*, *SWT1*, *ZKSCAN5*, *ZNF420*, *ZNF462*) (Supplementary Table S1). Interestingly, of the 73 genes, knockouts of 27 genes in mouse models led to functional consequences such as changes in the lean mass, fat mass and grip strength.

Of the 78 SNPs, 58 were identified as eQTL SNPs that correlated with expression of genes in various tissues including skeletal muscle and nervous system, indicating that they are likely to be functional and may influence multiple traits. Indeed, we found that risk alleles were also associated with other intermediate phenotypes of sarcopenia, namely tiredness, falls in the last year, low physical activity, and low bone mineral density. Furthermore, risk alleles were associated with neuroticism, time spent watching television, alcohol intake, smoking and poor diet (higher salt, white bread, and processed meat intake), whereas protective alleles were positively associated with serum testosterone, IGF1, and 25-hydroxyvitamin D levels, height, intelligence, cognitive performance, educational attainment, income, ground coffee drinking and healthier diet (muesli, cereal, wholemeal or wholegrain bread, potassium, magnesium, cheese, oily fish, protein, water, fruit, and vegetable intake).

This is in line with the previous studies stating that low educational attainment [41], neuroticism [42], low testosterone levels [43], short stature [44], high alcohol [45], processed meat [46] and salt [47] intake, sedentary behavior (such as watching television) [48], smoking and physical inactivity [49] are associated with an increased risk of sarcopenia or low muscle strength, whereas coffee, magnesium, potassium, protein, vitamin D, water, oil fish, fruits and vegetables intake [46,50,51,52,53,54] have protective effects against sarcopenia.

Future research is needed to test interventional strategies focusing on all these factors to evaluate improvement in muscle quality and quantity. Given that resistance exercise induces significant changes in the expression of 26 genes (out of 73 implicated genes) in human skeletal muscle compared to the pre-training state, our findings also partly explain beneficial effects of strength training in the prevention and treatment of sarcopenia [55].

The link of 78 SNPs with muscle strength and lean mass indicates that these markers may be important not only in the general population, but also in athletes. Indeed, of this panel of markers, some of protective alleles have been reported to be over-represented in elite sprinters (*E2F3* rs4134943 T, *FHL2* rs55680124 C, *GDF5* rs143384 G, *SLC39A8* rs13107325 C, and *ZNF568* rs1667369 A) [56] and elite strength athletes (*ADCY3* rs10203386 T, *ADPGK* rs4776614 C, *MMS22L* rs9320823 T and *ZKSCAN5* rs3843540 C) [57] compared to controls. Furthermore, *GBF1* rs2273555 G, *MLN* rs12055409 G, and *MMS22L* rs9320823 T alleles (all protective) were found to be positively associated with weightlifting performance [58,59]. One of markers (rs3734254) is located in the *PPARD* gene which is in high linkage disequilibrium (LD) with the *PPARD* rs2016520 SNP, which has been previously associated with endurance athlete status [60].

Several studies have investigated the association between the DNA polymorphisms and sarcopenia-related traits outside of the UK Biobank project using GWAS [61,62,63,64,65,66,67,68,69,70] or a candidate gene [71,72,73,74,75,76,77,78,79,80,81] approaches. Apart from the classical skeletal muscle traits (lean mass, absolute and relative skeletal muscle mass, skeletal muscle mass index, muscle thickness, anatomical cross-sectional area of muscle groups, etc.), genomic predictors of muscle fiber size (one of surrogate indicators of muscle mass) have been also studied [58,82,83,84,85].

Our study presents novel data of sarcopenia-related genetic markers. However, there are also limitations. Firstly, our findings were based on summary statistics of three different phenotypes which were discovered in the whole sample of the UK Biobank. To confirm the association between identified SNPs, sarcopenia and sarcopenic obesity, a case-control study (individuals with confirmed sarcopenia vs. individuals with normal muscle quality and quantity) is needed in independent studies. Second, our results were obtained using genomic data of European-ancestry individuals from the UK Biobank. Therefore, the set of 78 SNPs should be analyzed for association with sarcopenia in other populations as well before implementation in practice. We also recognise small sample size (*n* = 7) in the study of transcriptomic responses to a single-bout resistance exercise and encourage independent replication in larger cohorts.

## 5. Conclusions

In conclusion, we have identified and characterized 78 SNPs associated with sarcopenia and 55 SNPs with sarcopenic obesity that highlight shared genetic architecture between sarcopenia-related traits and lifestyle exposures.

We strongly suspect that many additional common polymorphisms, and probably rare mutations as well, will be shown to be associated with sarcopenia-related traits in due course. Thus, we suspect that the 78 polymorphisms we have identified constitute only a small fraction of the genetic factors that influence muscle strength, muscle mass and walking pace. However, looking to the future, when thousands of polymorphisms will be discovered that contribute to the variability in sarcopenia-related traits, the power of such information (in conjunction with standard measurement data) as a practical tool for clinicians will emerge.

## Figures and Tables

**Table 1:** The list of phenotypes used from the studies involving UK Biobank cohorts.

Phenotype	*p* Value	Number of Participants	Reference
Formation of initial list of SNPs associated with sarcopenia-related traits			
Appendicular lean mass (1059 SNPs)	<5 × 10^−9^	450,243	[20]
Maximal handgrip strength (16 SNPs)	<5 × 10^−8^	195,180	[17]
Relative handgrip strength (139 SNPs)	<5 × 10^−8^	334,925	[18]
Handgrip strength in older adults (15 SNPs)	<5 × 10^−8^	256,523	[19]
Self-reported walking pace (70 SNPs)	<5 × 10^−8^	450,967	[21]
Matching phenotypes to identify SNPs with pleiotropic effects			
Appendicular lean mass	<0.005	450,243	[20]
Appendicular lean mass in older adults	<5 × 10^−8^	181,862	[25]
Handgrip strength (left)	<0.005	359,704	[26]
Handgrip strength (right)	<0.005	359,729	[26]
Handgrip strength in older adults	<0.005	256,523	[19]
Usual walking pace	<0.005	358,974	[26]
Traits analysed for associations with the selected SNPs			
Body fat percentage	<0.005	354,628	[26]
Type 2 diabetes	<0.005	408,959	[26]
Heel bone mineral density	<0.005	426,824	[27]
Frequency of tiredness	<0.005	350,580	[26]
Self-reported tiredness	<0.005	108,976	[26]
Recent feelings of tiredness or low energy	<0.005	117,828	[26]
Falls in the last year	<0.005	360,344	[26]
Testosterone levels	<0.005	425,097	[28]
Insulin-like growth factor 1 (IGF1) levels	<0.005	435,516	[29]
25-hydroxyvitamin D levels	<0.005	417,580	[30]
Time spent watching television	<0.005	341,859	[26]
Vigorous physical activity	<0.005	261,055	[31]
Participation in strenuous sports	<0.005	359,263	[26]
Participation in other exercises	<0.005	359,263	[26]
Duration of moderate activity	<0.005	268,826	[26]
Duration of other exercises	<0.005	172,650	[26]
Moderate to vigorous physical activity levels	<0.005	377,234	[31]
Number of days/week of vigorous PA 10+ min	<0.005	344,084	[26]
Number of days/week of moderate PA 10+ min	<0.005	343,943	[26]
Alcohol intake frequency	<0.005	360,726	[26]
Current smoking/ever smoked	<0.005	360,797	[26]
Cheese intake	<0.005	352,458	[26]
Processed meat intake	<0.005	360,468	[26]
Oily fish intake	<0.005	359,340	[26]
Water intake	<0.005	333,363	[26]
Fruit (fresh or dried) intake	<0.005	329,134	[26]
Vegetable (cooked/salad/raw) intake	<0.005	350,404	[26]
Muesli intake	<0.005	299,898	[26]
Cereal intake	<0.005	345,019	[26]
Wholemeal or wholegrain bread intake	<0.005	348,424	[26]
White bread intake	<0.005	348,424	[26]
Ground (espresso, filter etc.) coffee intake	<0.005	283,449	[26]
Salt added to food	<0.005	360,954	[26]
Protein intake	<0.005	51,453	[26]
Potassium intake	<0.005	51,453	[26]
Magnesium intake	<0.005	51,453	[26]
Height	<0.005	458,235	[29]
Cognitive performance	<0.005	257,841	[32]
Intelligence	<0.005	269,867	[33]
Educational attainment	<0.005	357,549	[26]
Average total household income before tax	<0.005	311,028	[26]
Neuroticism	<0.005	380,506	[34]

SNPs, single nucleotide polymorphisms; PA, physical activity.

**Table 2:** DNA polymorphisms associated with sarcopenia-related traits, fat percentage and type 2 diabetes.

Gene/Near Gene	SNP	Protective Allele	Risk Allele	*p* Value				
				Handgrip Strength	Appendicular Lean Mass	Usual Walking Pace	Body Fat Percentage	Type 2 Diabetes
*GDF5*	rs143384	G	A	5.5 × 10^−46^	7.0 × 10^−319^	4.0 × 10^−8^	NS	NS
*POLD3*	rs72977282	T	A	7.4 × 10^−28^	9.3 × 10^−8^	3.6 × 10^−3^	NS	NS
*LCORL*	rs1472852	C	A	4.0 × 10^−24^	8.2 × 10^−135^	3.7 × 10^−4^	3.0 × 10^−5^	NS
*ADCY3*	rs10203386	T	A	1.6 × 10^−23^	1.7 × 10^−36^	3.3 × 10^−3^	2.4 × 10^−38^	NS
*DLEU1*	rs3116602	T	G	1.4 × 10^−21^	9.5 × 10^−155^	8.7 × 10^−4^	NS	NS
*AOC1*	rs6977416	A	G	6.7 × 10^−19^	1.4 × 10^−113^	7.7 × 10^−4^	5.6 × 10^−10^	NS
*SLC39A8*	rs13107325	C	T	2.0 × 10^−17^	3.9 × 10^−3^	1.8 × 10^−21^	5.0 × 10^−23^	1.1 × 10^−4^
*HLA-DRB1*	rs34415150	A	G	3.4 × 10^−17^	2.5 × 10^−17^	2.3 × 10^−5^	6.6 × 10^−4^	6.0 × 10^−17^
*HLA-DRB1*	rs2760975	G	A	4.6 × 10^−17^	1.7 × 10^−17^	1.6 × 10^−5^	8.4 × 10^−6^	6.9 × 10^−10^
*MLLT10*	rs1243182	C	T	3.5 × 10^−16^	2.6 × 10^−3^	3.9 × 10^−7^	6.5 × 10^−14^	NS
*PRRC2A*	rs2260051	A	T	1.0 × 10^−15^	5.2 × 10^−6^	2.6 × 10^−5^	3.3 × 10^−17^	1.7 × 10^−13^
*BTNL2*	rs2213581	T	C	1.1 × 10^−15^	4.3 × 10^−20^	6.3 × 10^−4^	3.3 × 10^−6^	3.2 × 10^−11^
*FKBPL*	rs41268905	G	A	2.4 × 10^−15^	8.9 × 10^−17^	4.0 × 10^−4^	1.3 × 10^−5^	NS
*ZBTB38*	rs2871960	C	A	4.0 × 10^−15^	2.2 × 10^−135^	1.0 × 10^−3^	NS	9.5 × 10^−5^
*ADCY3*	rs1056074	T	C	7.6 × 10^−15^	1.2 × 10^−11^	1.3 × 10^−3^	1.8 × 10^−17^	NS
*PML*	rs5742915	C	T	1.8 × 10^−14^	9.3 × 10^−39^	4.0 × 10^−5^	4.1 × 10^−8^	1.6 × 10^−3^
*POU6F2*	rs4549685	T	C	4.5 × 10^−14^	4.0 × 10^−7^	7.3 × 10^−5^	2.1 × 10^−12^	1.5 × 10^−4^
*HMGA2*	rs4338565	C	T	8.0 × 10^−14^	4.9 × 10^−151^	2.3 × 10^−4^	NS	1.4 × 10^−3^
*HLA-DRB1*	rs113315602	A	C	1.4 × 10^−12^	1.0 × 10^−5^	5.4 × 10^−4^	3.5 × 10^−6^	NS
*WWP2*	rs4985445	A	G	1.4 × 10^−12^	3.3 × 10^−20^	1.2 × 10^−5^	4.6 × 10^−18^	1.7 × 10^−5^
*MTCH2*	rs11039324	G	A	3.5 × 10^−12^	7.3 × 10^−26^	3.9 × 10^−15^	9.0 × 10^−38^	3.2 × 10^−6^
*HLA-DRB5*	rs117108573	C	T	3.6 × 10^−11^	1.1 × 10^−4^	7.4 × 10^−4^	3.4 × 10^−4^	NS
*GBF1*	rs2273555	G	A	4.1 × 10^−11^	8.1 × 10^−5^	8.2 × 10^−5^	NS	NS
*SFMBT1*	rs62253653	G	A	9.5 × 10^−11^	3.0 × 10^−3^	1.0 × 10^−7^	4.0 × 10^−3^	1.2 × 10^−3^
*JARID2*	rs2237149	A	C	7.5 × 10^−10^	4.5 × 10^−5^	1.8 × 10^−4^	6.4 × 10^−6^	NS
*ADPGK*	rs4776614	C	G	1.9 × 10^−9^	8.4 × 10^−6^	1.4 × 10^−4^	5.6 × 10^−13^	NS
*JUND*	rs7249	T	C	2.0 × 10^−9^	1.2 × 10^−6^	2.6 × 10^−4^	5.8 × 10^−13^	NS
*KIF1B*	rs3903151	G	A	2.6 × 10^−9^	1.3 × 10^−16^	3.0 × 10^−4^	1.2 × 10^−4^	2.9 × 10^−3^
*SWT1*	rs10797999	T	C	3.0 × 10^−9^	1.4 × 10^−5^	1.4 × 10^−8^	2.5 × 10^−4^	NS
*FOXP1*	rs4677611	T	C	3.2 × 10^−9^	2.4 × 10^−4^	2.2 × 10^−3^	2.3 × 10^−5^	NS
*SOX5*	rs11047225	C	T	8.5 × 10^−9^	4.3 × 10^−10^	2.2 × 10^−3^	NS	NS
*NCOA1*	rs77012907	A	G	1.2 × 10^−8^	1.2 × 10^−13^	5.8 × 10^−4^	2.9 × 10^−15^	NS
*MMS22L*	rs9320823	T	C	1.4 × 10^−8^	7.9 × 10^−10^	1.1 × 10^−6^	3.9 × 10^−22^	1.5 × 10^−4^
*ZKSCAN5*	rs3843540	C	T	2.3 × 10^−8^	5.8 × 10^−6^	1.1 × 10^−4^	4.0 × 10^−9^	NS
*MLN*	rs12055409	G	A	3.5 × 10^−8^	3.6 × 10^−3^	1.8 × 10^−3^	3.2 × 10^−7^	4.4 × 10^−4^
*FOXP1*	rs830643	A	G	4.0 × 10^−8^	9.7 × 10^−7^	2.9 × 10^−7^	5.7 × 10^−8^	9.3 × 10^−6^
*GADD45G*	rs1329733	A	G	4.3 × 10^−8^	2.2 × 10^−4^	6.4 × 10^−5^	9.0 × 10^−13^	NS
*IL11*	rs4252548	C	T	6.2 × 10^−8^	4.8 × 10^−35^	2.6 × 10^−3^	NS	NS
*COMMD4*	rs11636600	G	A	6.8 × 10^−8^	5.0 × 10^−13^	9.7 × 10^−11^	NS	NS
*HABP4*	rs6477489	C	A	7.2 × 10^−8^	5.2 × 10^−59^	3.7 × 10^−3^	1.1 × 10^−3^	NS
*GLCCI1*	rs12702693	T	C	1.8 × 10^−7^	6.6 × 10^−20^	1.4 × 10^−3^	NS	NS
*H1FX*	rs4073154	G	A	2.5 × 10^−7^	1.9 × 10^−33^	3.3 × 10^−8^	NS	NS
*CEP192*	rs1786263	G	T	3.1 × 10^−7^	1.0 × 10^−22^	2.3 × 10^−3^	5.5 × 10^−7^	NS
*PPARD*	rs3734254	T	C	5.2 × 10^−7^	2.3 × 10^−33^	5.6 × 10^−6^	NS	NS
*ZNF568*	rs1667369	A	C	1.3 × 10^−6^	1.5 × 10^−10^	2.7 × 10^−8^	1.7 × 10^−3^	NS
*SERPINA1*	rs28929474	T	C	3.5 × 10^−6^	1.1 × 10^−14^	3.4 × 10^−4^	NS	2.6 × 10^−3^
*NMT1*	rs2301597	C	T	4.6 × 10^−6^	4.8 × 10^−33^	2.7 × 10^−9^	1.3 × 10^−8^	NS
*PIEZO1*	rs2968478	T	G	5.1 × 10^−6^	5.6 × 10^−14^	2.8 × 10^−3^	NS	NS
*CELF4*	rs12962050	A	G	6.8 × 10^−6^	1.5 × 10^−14^	4.8 × 10^−4^	2.1 × 10^−3^	NS
*BCKDHB*	rs9350850	C	T	7.5 × 10^−6^	2.9 × 10^−24^	1.3 × 10^−3^	NS	NS
*E2F3*	rs4134943	T	C	9.9 × 10^−6^	2.0 × 10^−8^	4.9 × 10^−9^	6.7 × 10^−5^	1.2 × 10^−5^
*BTRC*	rs10883618	A	G	2.6 × 10^−5^	1.8 × 10^−4^	4.1 × 10^−9^	1.5 × 10^−6^	4.6 × 10^−3^
*LIN28A*	rs4274112	A	G	2.8 × 10^−5^	2.5 × 10^−28^	8.5 × 10^−4^	6.8 × 10^−4^	3.3 × 10^−4^
*ZNF420*	rs62108897	C	A	6.3 × 10^−5^	7.7 × 10^−19^	4.7 × 10^−3^	7.1 × 10^−4^	NS
*JUND*	rs10686842	TAAA	T	6.4 × 10^−5^	7.4 × 10^−24^	3.7 × 10^−4^	1.6 × 10^−19^	NS
*DIPK1A*	rs12733767	C	T	6.8 × 10^−5^	9.4 × 10^−14^	9.0 × 10^−5^	3.2 × 10^−3^	NS
*IGF2BP3*	rs34776209	C	T	6.9 × 10^−5^	1.8 × 10^−47^	8.4 × 10^−4^	NS	NS
*XPO4*	rs7321635	A	C	9.0 × 10^−5^	2.6 × 10^−11^	4.5 × 10^−4^	NS	NS
*FHL2*	rs55680124	C	T	1.1 × 10^−4^	4.2 × 10^−4^	2.6 × 10^−9^	6.0 × 10^−8^	2.3 × 10^−7^
*VCAN*	rs115912456	G	A	1.8 × 10^−4^	3.7 × 10^−34^	1.8 × 10^−3^	3.5 × 10^−14^	NS
*RBL2*	rs72801843	A	T	1.9 × 10^−4^	8.8 × 10^−52^	8.9 × 10^−6^	NS	4.5 × 10^−5^
*NPPC*	rs73000823	C	T	2.3 × 10^−4^	1.7 × 10^−15^	3.7 × 10^−4^	1.4 × 10^−5^	NS
*MYO1C*	rs9905106	T	C	3.5 × 10^−4^	7.5 × 10^−14^	3.9 × 10^−3^	4.4 × 10^−5^	NS
*CDKAL1*	rs745771286	G	GA	3.9 × 10^−4^	2.7 × 10^−11^	4.8 × 10^−5^	4.7 × 10^−5^	NS
*GIP*	rs4794005	A	G	4.3 × 10^−4^	8.8 × 10^−15^	1.1 × 10^−3^	4.9 × 10^−4^	3.6 × 10^−4^
*NCL*	rs10202701	T	C	4.3 × 10^−4^	3.1 × 10^−33^	2.0 × 10^−3^	2.3 × 10^−5^	NS
*SOCS5*	rs62136933	A	G	5.7 × 10^−4^	9.0 × 10^−32^	2.1 × 10^−4^	3.8 × 10^−11^	NS
*CAMKMT*	rs11893991	A	G	6.0 × 10^−4^	2.3 × 10^−9^	2.4 × 10^−3^	NS	NS
*RIN3*	rs117068593	T	C	6.0 × 10^−4^	8.8 × 10^−62^	3.9 × 10^−3^	3.8 × 10^−10^	NS
*JMJD1C*	rs7924036	T	G	8.9 × 10^−4^	1.2 × 10^−5^	1.2 × 10^−13^	NS	NS
*TRIB1*	rs4870941	G	C	1.3 × 10^−3^	1.1 × 10^−39^	2.1 × 10^−3^	NS	NS
*SDCCAG8*	rs2994330	T	G	2.4 × 10^−3^	6.6 × 10^−12^	9.6 × 10^−5^	3.0 × 10^−4^	NS
*NYAP2*	rs2054079	T	C	3.0 × 10^−3^	3.9 × 10^−4^	4.3 × 10^−9^	NS	NS
*MAML3*	rs57800857	C	A	3.5 × 10^−3^	4.0 × 10^−7^	6.4 × 10^−11^	1.4 × 10^−13^	2.7 × 10^−4^
*PITX1*	rs4976261	G	C	3.6 × 10^−3^	8.7 × 10^−43^	1.7 × 10^−3^	NS	NS
*PKDCC*	rs3035165	T	TTA	3.7 × 10^−3^	6.9 × 10^−14^	1.0 × 10^−3^	3.0 × 10^−8^	NS
*HTT*	rs362307	C	T	3.9 × 10^−3^	2.5 × 10^−7^	1.1 × 10^−9^	2.8 × 10^−9^	1.3 × 10^−6^
*ZNF462*	rs902144	C	G	4.0 × 10^−3^	7.2 × 10^−13^	4.0 × 10^−4^	1.7 × 10^−3^	NS

NS, not significant (*p* > 0.005). SNP, single nucleotide polymorphism. Handgrip strength: any type of handgrip strength (maximal or relative handgrip strength, left or right handgrip strength, handgrip strength in older adults) with the lowest *p* value. Protective allele: allele associated with greater strength, lean mass, and walking speed and low risk of obesity and T2D. Risk allele: allele associated with lower strength, lean mass, walking speed and high risk of obesity and T2D.

**Table 3:** Stratification of European individuals by the number of risk alleles.

Trait	Disease Risk and Number of Risk Alleles				
	Low	Below Average	Average	Above Average	High
Sarcopenia (78 SNPs)	58–68	69–72	73–76	77–80	81–95
Sarcopenic obesity (55 SNPs)	37–47	48–50	51–53	54–57	58–70
Sarcopenic diabesity (21 SNPs)	10–16	17–18	19–20	21–22	23–30

SNPs, single nucleotide polymorphisms. The ranges of the number of risk alleles in European individuals with different degrees of the diseases risks is shown.

## Data Availability

The data presented in this study are publicly available online at https://genetics.opentargets.org (accessed on 27 December 2022).

## Supplementary Materials

The following supporting information can be downloaded at: https://www.mdpi.com/article/10.3390/nu15030758/s1, Table S1: Association of top sarcopenia-related SNPs and implicated genes with health-related traits, gene expression and responses to resistance training.
